# An assessment of serial co-cultivation approach for generating novel *Zymomonas mobilis* strains

**DOI:** 10.1186/s13104-020-05261-5

**Published:** 2020-09-07

**Authors:** Katsuya Fuchino, Per Bruheim

**Affiliations:** Department of Biotechnology and Food Science, Norwegian University of Science and Technology, Trondheim, Norway

**Keywords:** *Zymomonas mobilis*, Ethanol production, Adaptive evolution, Co-culture, Genomic stability

## Abstract

**Objective:**

The alphaproteobacterium *Zymomonas mobilis* is an efficient ethanol producer, and *Z. mobilis*-based biorefinery shows great potential for biofuel production. Serial co-cultivation is an emerging approach that promotes inter-species interactions which can improve or rewire the metabolic features in industrially useful microorganisms by inducing frequent mutations. We applied this method to assess if it improves or rewires the desirable physiological features of *Z. mobilis*, especially ethanol production.

**Results:**

We performed serial co-culture of *Z. mobilis* with the baker’s yeast, *Saccharomyces cerevisiae*. We observed filamentation of *Z. mobilis* cells in the co-culture, indicating that the *Z. mobilis* cells were exposed to stress due to the presence of a competitor. After 50 times of serial transfers, we characterized the generated *Z. mobilis* strains, showing that long term co-culture did not drive significant changes in either the growth or profile of excreted metabolites in the generated strains. In line with this, whole genome sequencing of the generated *Z. mobilis* strains revealed only minor genetic variations from the parental strain. 50 generations of *Z. mobilis* monoculture did not induce morphological changes or any significant genetic variations. The result indicates that the method needs to be carefully optimized for *Z. mobilis* strain improvement.

## Introduction

The alphaproteobacterium *Z. mobilis* is the best bacterial ethanol producer endowed with unique physiological features [1]. Therefore, *Z. mobilis* based-biorefinery is a promising biofuel production system at an industrial scale [2]. Despite its efficient ethanol production capacity, there are challenges in employing *Z. mobilis* as a biocatalyst. For example, *Z. mobilis* consumes a limited range of feedstock as substrate [2]. It is also known to be sensitive to certain abiotic stress, such as acetic acid toxicity [2, 3]. Yet, recent advances in *Z. mobilis* metabolic engineering have overcome these drawbacks, advancing its potential use for environmentally-friendly biorefining [2, 3].

In addition to ethanol production, rewiring metabolic pathways to produce other useful compounds has lately been explored in *Z. mobilis* [3–7]. This is to utilize and exploit its intrinsic capacity of fast catabolism that comes with small biomass accumulation [1]. Considering that its prolific potential is expanding, a novel approach to engineer or generate desirable *Z. mobilis* strains should be assessed.

Co-culture based adaptive evolution involves mixing several species in the same culture to stimulate inter-species interactions, this novel and recent approach improves the physiological features of industrially beneficial microorganisms by inducing frequent mutations [8]. For example, serial co-culture of *Candida glabrata* and *Pichia kudriavzevii* significantly influenced the growth and fermentation profile of co-evolved strains [9]. The evolved strains conferred altered chemical complexity in produced wine [9]. Zhou et al. showed that long term bacterial-yeast competition induced chromosomal arrangements in the yeast, rendering stress-tolerance, altered metabolism and other physiological features in the yeast *Lachancea kluyveri* [10]. In addition to promoting mutation, co-culture has also been shown to promote the production of particular metabolites in *Streptomyces* species, which were otherwise not expressed in pure monoculture [11, 12]. Thus, inter-species interaction stimulates expression of cryptic genes. [11–13]. Such a response might also bias mutational events if the cryptic genes were continuously expressed during serial co-culture.

In addition to gaining potentially desirable traits, another advantage of the serial co-culture based adaptation method is to shed light on the understanding of basic ecological aspects of species interaction [14, 15]. Given that *Z. mobilis* ecology and its natural habitat is yet rather enigmatic [16], the approach should be worth being examined in this regard as well.

In the present work, we adopted serial co-culture of *Z. mobilis* mixed with baker’s yeast *Saccharomyces cerevisiae* for strain generation. The aim of study was to see if *Z. mobilis* changes or rewires its ethanologenic features through competition and interaction with *S. cerevisiae* through serial co-cultivation.

## Main text

### Method

#### Cultivation

*Z. mobilis* Zm6 and *S. cerevisiae* strain CEN.PK113-7D strain were used for adaptive evolution by co-culture. Cells were grown in growth medium containing glucose (20 g/L or 100 g/L), yeast extract (5 g/L), NH_4_SO_4_ (1 g/L), KH_2_PO_4_ (1 g/L) and MgSO_4_ (0.5 g/L). The growth medium was flushed with nitrogen gas prior to culture. The co-culture was grown at 30 °C in a tightly capped test tube with shaking at 200 rpm throughout the study.

For serial co-culture, 10 μL of Zm6 overnight anaerobic culture and *S. cerevisiae* anaerobic culture were used as starters. The mixed cultures typically spent all nutrients within a day. The fully grown cultures (10 μL) were transferred to fresh identical medium the following day and the co-culture continued. After the transfer was repeated 5 times, we observed that *Z. mobilis* competes out in the co-culture under the condition, as shown by viable counts of the co-culture. To maintain balanced co-culture, we inoculated additional *S. cerevisiae* overnight culture 40 μL, in addition to 10 μL of previous co-culture, upon each transfer from 6th round.

For *Escherichia coli* co-culture with *Z. mobilis*, *E. coli* strain K12 grown in LB medium under aerobic condition was used as a starter. We started co-culture with mixing 10 μL of fully grown monocultures of *Z. mobilis* and *E. coli*. During the experiments, we learned that inoculating 10 μL of fully grown co-culture of previous round with 20 μL of fully grown *E. coli* monoculture upon a transfer gave a good balance for continuation of co-cultures, which we performed for all transfer. All lines were replicated for whole passages.

After the serial co-culture of 50 transfers, cells were streaked out on the solid identical medium. Isolated pure strains are designated as follows, Zs100: *Z. mobilis* Zm6 derived strain obtained from last round of *Z. mobilis* vs *S. cerevisiae* serial co-culture supplemented with glucose 100 g/L. Zs100R is obtained from a parallel replicate of *Z. mobilis* vs *S. cerevisiae* glucose 100 g/L. Zs20: Zm6 derived strain obtained from the last round of serial co-culture with *S. cerevisiae* supplemented with 20 g/L glucose, and Zs20R was obtained from a last culture of parallel run of Zs20. Similarly, Ze20 designates *Z. mobilis* strain obtained from last round of *Z. mobilis* vs *E. coli* co-culture with 20 g/L glucose, and Ze20R as a parallel replicate. As a control, the 50 times serial transfer of *Z. mobilis* monocultures grown in the same complex medium with 20 g/L glucose or 100 g/L glucose were performed. Z20 designates *Z. mobilis* strain obtained from a last round of *Z. mobilis* monoculture with 20 g/L glucose, and Z100 designates *Z. mobilis* strain obtained from a last round of monoculture with 100 g/L glucose.

#### Characterization of growth and ethanol production

Growth profiles of all strains were analyzed using flat bottom 96-well microplate in plate-reader spark 20 M (Tecan) by measuring its absorbance at 600 nm. Overnight anaerobic monoculture was used as an inoculum. Temperature control was set at 30 °C. Three technical replicates were repeated for each time point. The used medium in plate reader was same as for co-culture, with supplement of glucose 20 g/L. We also measured growth profiles in test tubes under anaerobic condition using spectrophotometer (VWR), showing similar trends from microplate experiments.

Acetate, lactate, ethanol and glucose in the overnight monoculture of generated *Z. mobilis* strains were measured using Waters 2695e Alliance HPLC (Waters) with Hi-plex column (300 × 7.7 mm, Agilent). The spent medium of anaerobic overnight culture in the test tube was analyzed for HPLC analysis. The collected supernatant was filtered through 0.2 μm Supor^®^ polyether sulfone membrane (PALL) before the analysis. HPLC analysis was run under the condition; 0.05 M sulfuric acid as mobile phase at a flow rate of 0.8 mL/minute. External standard curve was used for converting obtained peaks to concentration of analytes.

#### Microscopy

Growing sample was directly mounted on Phosphate-buffered saline (PBS)-agarose pad before imaging. Zeiss Axio Imager Z2 microscope (ZEISS) equipped with camera Axiocam MR R3 (ZEISS) was used for capturing phase contrast images. Software ZEN 3.1 (ZEISS) was used for image analysis.

#### Whole genome sequencing

Total DNA was extracted by combining a lysozyme treatment [17] and D-neasy blood tissue kit (Qiagen). Whole genome of the co-cultured *Z. mobilis* strains was sequenced by GATC re-sequencing service (INVIEW Genome sequencing). The reference sequence was our lab stock Zm6 strain which was previously sequenced [18, 19].

### Results

**Fig. 1:**
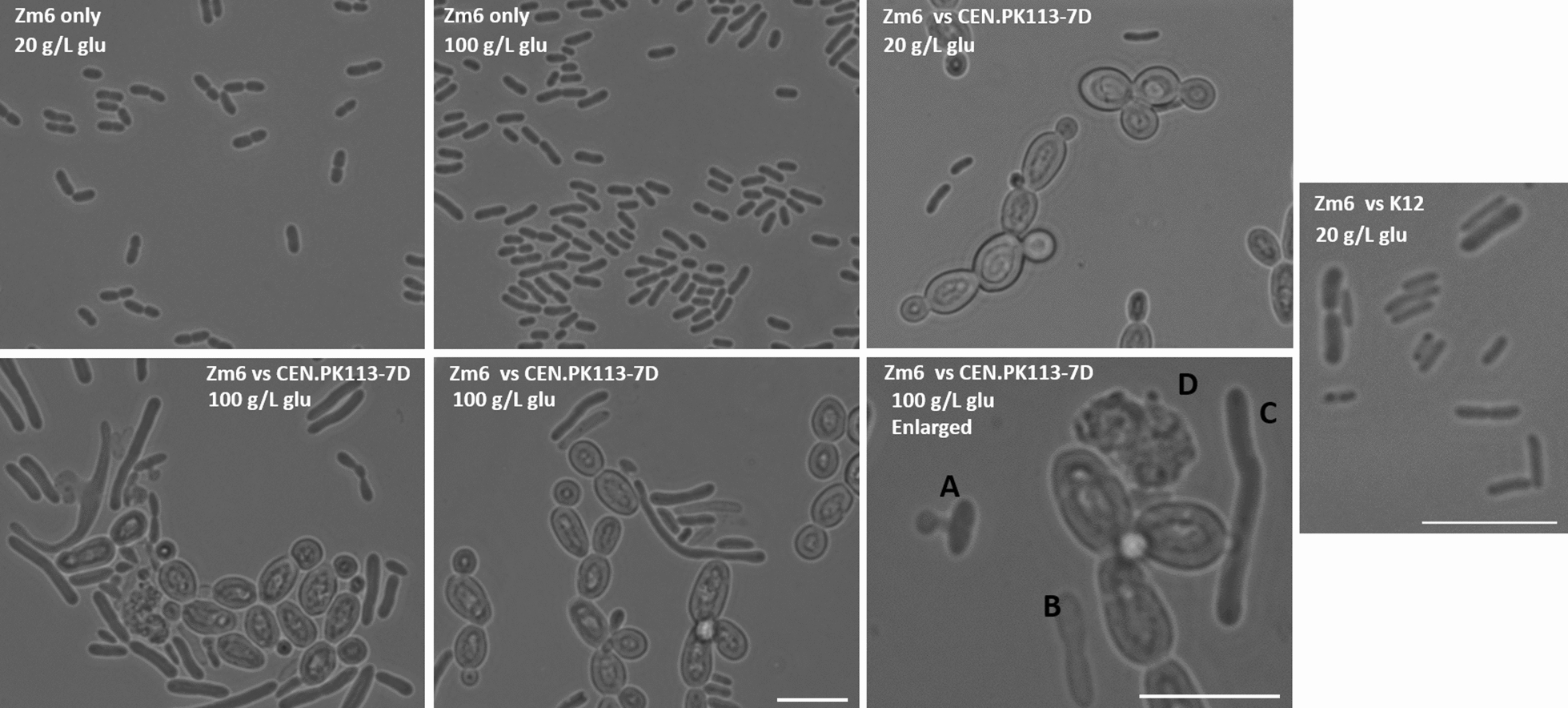
Phase contrast images of *Z. mobilis* monoculture and co-culture with *S. cerevisiae* strain CEN.PK113-7D or *E. coli* strain K12. Used strains and conditions are as shown in white texts in the images. Abbreviation glu stands for glucose in the medium. It is to be noted that *Z. mobilis* strain in the co-culture vs yeast with 100 g/L glucose formed elongated filamentous structure, while other conditions did not induce such drastic morphological changes in *Z. mobilis* cells. Right-bottom panel and Zm6 vs K12 panel are enlarged images with scale bar 10 μm, while all other panel sizes are corresponding with the scale bar 10 μm, found in the bottom-middle panel. Black **a** in the right-bottom panel indicates bursting cell with membrane protrusion from the cytoplasm. **b** The lysed cell exhibiting lighter phase contrast than that of the live cells. **c** Elongated *Z. mobilis* cell. **d** Dead yeast cell

In order to generate novel *Z. mobilis* strains, we assessed the co-culturing based adaptive evolution. We chose serial transfer instead of continuous chemostat co-culture, for the simplicity of method, and baker’s yeast *S. cerevisiae* as a competitor since it also produces ethanol and likely share an ecological niche with *Z. mobilis*, although ethanol production pathway differs between two species. During the pilot experiment, we observed that many *Z. mobilis* cells showed abnormal cell shape in the co-culture with *S. cerevisiae* supplemented with 100 g/L glucose (Fig. 1). Most of *Z. mobilis* cells formed filamentous structures which can be up to 10 times longer than regular cell (Fig. 1, shown as C in the lower-right panel). Some of the *Z. mobilis* cells were burst or exhibiting a membrane protrusion from cell (Fig. 1, shown as A in lower-right panel) and several cells were found dead as shown by lighter phase contrast (Fig. 1b). Dead *S. cerevisiae* cells were also observed (Fig. 1, shown as D in lower-right panel). It should be noted that these morphological changes or death of *Z. mobilis* cells were not observed in the *Z. mobilis* monoculture using the same growth condition. Bacterial filamentation is often an adaption to stress [20]. Thus, we found the observed phenomenon as a good indication that the Zm6 cells were interacting and competing with *S. cerevisiae*. This prompted us to expect that frequent mutations should arise in the *Z. mobilis* cells to gain advantages for the competition during serial co-culture. In the co-culture with *E. coli*, obvious morphological changes of *Z. mobilis* and *E. coli* was not observed.

We then performed serial transfer of each mixed culture for 50 passages. For co-culture with *S. cerevisiae*, we made two lines of culture supplemented with 20 g/L or 100 g/L glucose. For *E. coli* co-culture, we performed cultivation only with 20 g/L glucose. All co-cultures were run with parallel replicates, resulting in 6 lines of serial co-cultures. In addition, we performed serial transfer of two monocultures of *Z. mobilis* strains for 50 passages as controls.

**Fig. 2:**
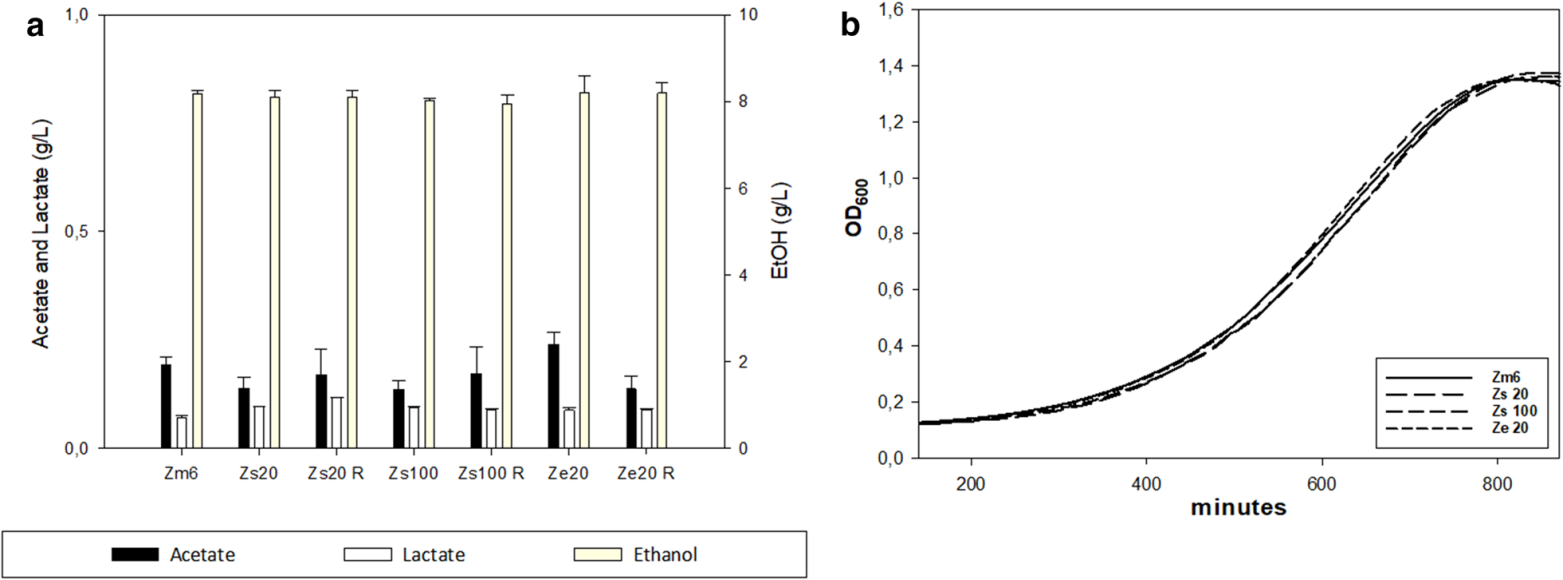
Characterization of *Z. mobilis* strains that generated from serial co-culture-based laboratory adaptation. **a** Total production of acetate, lactate and ethanol from the overnight monoculture of the generated *Z. mobilis* strains and the parental strain Zm6. **b** Growth profiles of monoculture *Z. mobilis* strain obtained in this study and the parental strain Zm6 in the complex medium with glucose 20 g/L. *Z. mobilis* strain designations are as follows; Zs100; *Z. mobilis* strain obtained from the last round of *Z. mobilis* vs *S. cerevisiae* serial co-culture with glucose 100 g/L, Zs100R; obtained from a parallel replicate of *Z. mobilis* vs *S. cerevisiae* glucose 100 g/L, Zs20; *Z. mobilis* strain obtained from the last round of serial co-culture with *S. cerevisiae* supplemented with 20 g/L glucose, Zs20R; *Z. mobilis* strain from a last culture of parallel run of Zs20, Ze20; *Z. mobilis* strain obtained from last round of *Z. mobilis* vs *E. coli* co-culture with 20 g/L glucose, and Ze20R as a parallel replicate of Ze20, Z20 designates *Z. mobilis* strain obtained from a last round of *Z. mobilis* monoculture with 20 g/L glucose, and Z100 designates *Z. mobilis* strain obtained from a last round of monoculture with 100 g/L glucose. Generation time for actively growing *Z. mobilis* strains in the plate reader was as follows; 175 min for Zm6, 177 min for Zs20, 173 min for Zs100 and 179 min for Ze20. Note that there were no significant differences in ethanol production and growth profiles between strains. Error bars; standard deviations from 3 independent measurements

After 50 transfers of serial co-cultures, we characterized growth profile and ethanol production by monoculture of obtained *Z. mobilis* strains from last batch of long-term co-cultures. It was to be noted that the elongated morphology observed during serial co-culture was not retained in *Z. mobilis* cells grown in monoculture, suggesting that filamentation was due to stress. The HPLC and growth curve data showed that excreted production and growth profiles by obtained strains were nearly identical to those by parental strain (Fig. 2). This was somewhat contrary to what we anticipated, especially in the *Z. mobilis* cells with *S. cerevisiae* in 100 g/L glucose, since the *Z. mobilis* cells in that condition underwent long cultivation under constant stress, which could have influenced its metabolism or growth profile for better survival. This result is in sharp contrast to our previous experience with *Z. mobilis* adaptive evolution against salt stress, in which 10-20 passages of serial transfer drove the strains to gain adapted phenotype of two times better total ethanol production under the saline condition than that of the parental strain [19]. The obtained result here showed that the competition with yeast or *E. coli* in the co-culture did not influence *Z. mobilis* ethanologenic physiology.

Next, to see if there are mutations not reflected in the measured phenotypes, we sequenced whole genome of the obtained *Z. mobilis* strains. The analysis revealed that several mutations in the ORF regions of the generated strains, compared to the reference genome [18]. Yet, it was not clear how the mutations could relate to advantage in the species interaction. Interestingly, *Z. mobilis* strain Zs100R did not show any mutation in the ORF regions, despite long-term constant competition with *S. cerevisiae*. The strain Z20 obtained from serial-monoculture did not show any mutations either. This suggests that genomic stability was very high in *Z. mobilis* during the serial co-culture and monoculture.

### Discussion

Even though co-culture based adaptive evolution has shown to be a powerful tool for several bacterial and yeast species, we recommend that the method should be carefully fine-tuned for *Z. mobilis*. Our data suggest that serial co-culture methods utilizing species-interaction did not drive mutations in the genome of *Z. mobilis* strains in our experimental setting. Although the presence of *S. cerevisiae* induced stressed shape of *Z. mobilis*, the genome content of *Z. mobilis* cells was found almost intact (Table 1). Perhaps, utilizing more ‘stressful’ organism to *Z. mobilis* in the culture might drive the need of better fitness in *Z. mobilis* and thus improve an outcome of the method. Alternatively, *Z. mobilis* genomic stability might be very stable during species interaction. *Z. mobilis* was previously suggested to possess polyploidy [21, 22], and that could render genetic stability during stress responses by frequent homologous recombination. Confirmation of polyploidy and identification of copy numbers of chromosome in *Z. mobilis* cells should be useful knowledge for *Z. mobilis* research. Interestingly, recent report suggests that *Z. mobilis* is not involved in pair-wise cross feeding with other bacteria [23], implying that *Z. mobilis* does not cooperate with other microbes for its survival.

**Table 1:** A table of list of mutations found in generated *Z. mobilis* strains. Note that all the strains were obtained from the last round of serial co-culture or monoculture. NA stands for not available

Strain	Serial co-culture method	Position of the genome	Referencee	Mutation	Gene	Annotation
Zs100	*Z. mobilis* vs *S. cerevisiae* in the medium with 100 g/L glucose	87016	GA	G	ZZ6_0076	Hypothetical protein
Zs100R	*Z. mobilis* vs *S. cerevisiae* in the medium with 100 g/L glucose, replicate	NA		NA		
Zs20	*Z. mobilis* vs *S. cerevisiae* in the medium with 20 g/L glucose	NA		NA		
Zs20R	*Z. mobilis* vs *S. cerevisiae* in the medium with 20 g/L glucose, replicate	114095	C	T	ZZ6_0103	Hypothetical protein
Ze20	*Z. mobilis* vs *E. coli* in the medium with 20 g/L glucose	1195056	C	T	ZZ6_1040	TonB-dependent receptor
Ze20R	*Z. mobilis* vs *E. coli* in the medium with 20 g/L glucose, replicate	1185646	A	AGGCTCAG	ZZ6_1032	Heptose-I-phosphate ethanolaminephosphotransferase
Z20	*Z. mobilis* monoculture in the medium with 20 g/L glucose	NA		NA		
Z100	*Z. mobilis* monoculture in the medium with 100 g/L glucose	531639	G	A	ZZ6_4600	UDP-N-acetylmuramate–l-alanine ligase

## Limitation

Apart from growth curves and excreted metabolites profiles, there might be hidden phenotypes in generated strains that we did not detect. Changes in the genome of *S. cerevisiae* after the serial co-culture with *Z. mobilis* was not followed in this study. An investigation on *S. cerevisiae* strains that are co-cultured for long terms with *Z. mobilis* under appropriate experimental settings might be a future line of investigation.

## Data Availability

The datasets, sequenced genomes of the studied strains, and strains generated from the present work are available from the corresponding author upon on a reasonable request.

## Abbreviations

HPLC: High-pressure liquid chromatography
OD: Optical Density
ORF: Open Reading Frame
